# Fabrication of Semiconductor ZnO Nanostructures for Versatile SERS Application

**DOI:** 10.3390/nano7110398

**Published:** 2017-11-19

**Authors:** Lili Yang, Yong Yang, Yunfeng Ma, Shuai Li, Yuquan Wei, Zhengren Huang, Nguyen Viet Long

**Affiliations:** 1 State Key Laboratory of High Performance Ceramics and Superfine Microstructures, Shanghai Institute of Ceramics, Chinese Academy of Sciences, 1295 Dingxi Road, Shanghai 200050, China; llyang@student.sic.ac.cn (L.Y.); mayunfeng@student.sic.ac.cn (Y.M.); lishuai@student.sic.ac.cn (S.L.); weiyq@shanghaitech.edu.cn (Y.W.); zhrhuang@mail.sic.ac.cn (Z.H.); 2 Graduate University of Chinese Academy of Sciences, No. 19(A) Yuquan Road, Beijing 100049, China; 3 Ceramics and Biomaterials Research Group, Ton Duc Thang University, Ho Chi Minh City 800010, Vietnam; nguyenvietlong@tdt.edu.vn

**Keywords:** ZnO nanostructures, SERS, versatile substrates, preparation methods, meaningful averaged EFs

## Abstract

Since the initial discovery of surface-enhanced Raman scattering (SERS) in the 1970s, it has exhibited a huge potential application in many fields due to its outstanding advantages. Since the ultra-sensitive noble metallic nanostructures have increasingly exposed themselves as having some problems during application, semiconductors have been gradually exploited as one of the critical SERS substrate materials due to their distinctive advantages when compared with noble metals. ZnO is one of the most representative metallic oxide semiconductors with an abundant reserve, various and cost-effective fabrication techniques, as well as special physical and chemical properties. Thanks to the varied morphologies, size-dependent exciton, good chemical stability, a tunable band gap, carrier concentration, and stoichiometry, ZnO nanostructures have the potential to be exploited as SERS substrates. Moreover, other distinctive properties possessed by ZnO such as biocompatibility, photocatcalysis and self-cleaning, and gas- and chemo-sensitivity can be synergistically integrated and exerted with SERS activity to realize the multifunctional potential of ZnO substrates. In this review, we discuss the inevitable development trend of exploiting the potential semiconductor ZnO as a SERS substrate. After clarifying the root cause of the great disparity between the enhancement factor (EF) of noble metals and that of ZnO nanostructures, two specific methods are put forward to improve the SERS activity of ZnO, namely: elemental doping and combination of ZnO with noble metals. Then, we introduce a distinctive advantage of ZnO as SERS substrate and illustrate the necessity of reporting a meaningful average EF. We also summarize some fabrication methods for ZnO nanostructures with varied dimensions (0–3 dimensions). Finally, we present an overview of ZnO nanostructures for the versatile SERS application.

## 1. Introduction

Surface-enhanced Raman scattering (SERS) has attracted great interest as a real-time surface analysis technique with many advantages [1,2,3] such as ultra-sensitivity, non-destructivity, “fingerprint” ability, and low requirement for samples. It was first observed by Fleishman [4] in 1974 when his research group found that the Raman signal of pyridine on rough Ag electrode was abnormally enhanced by 10^5^–10^6^. In 1977, the SERS phenomenon was first disclosed by Albrecht [5] and Jeanmaire [6]. Subsequently, scientists from various disciplines began to study the nature and mechanism of SERS and further implement its application. Until now, many achievements have been made and SERS has been successfully applied in many domains, including biomolecule [7,8] and pesticide detection [9,10,11], molecule imaging [12], identification of cancer cells [13], dynamic study of catalytic reactions [14,15], terrorist threat detection [16,17], food safety [18], etc. A prerequisite for SERS to come into play is the choice, design, and preparation of the substrate materials. The SERS activities of substrates are usually evaluated by an important parameter called the enhancement factor (EF).

The earliest developed and the most widely used SERS substrate materials are noble metallic nanostructures (Au, Ag, Cu) [19,20]. They have a unique superiority as SERS substrates, namely their ultra-high SERS sensitivity with a maximal EF of 10^14^–10^15^; thus, they can even be used for single-molecule detection [21,22,23,24,25]. Nevertheless, their disadvantages, such as difficulties in fabricating highly-uniform nanostructures at low cost [26], instability problems including easy aggregation and oxidation during application [27], as well as a limited number of noble metals with excellent SERS activity have hindered the development and wide application of these noble metal substrates.

After being confirmed to have SERS activity themselves [28,29], semiconductors [30,31,32] are gradually being exploited as promising candidates for SERS substrates owing to their outstanding advantages [33,34,35] such as abundant active substrates, diverse and mature synthetic techniques at low cost, controllable band structure and photoelectrical properties, and high chemical stability and biocompatibility when compared with noble metals. Additionally, synergistic collaboration between SERS and other properties ranging from photocatalysis [36], magnetism [37], to gas-, bio-, and chemo-sensing [38,39] can be realized in semiconductors.

It is well known that ZnO is a wide band gap (~3.3 eV) metallic oxide semiconductor with distinctive physical, chemical, and photoelectric properties [40,41,42]. At the same time, ZnO nanomaterials can be grown into numerous morphologies, including nanospheres, nanowires, nanorods, nanoneedles, nanocones, nanobelts, nanocombs, nanorings, nanosprings, and nanocages [42,43,44]. It is one of the most common and versatile semiconductors, with a wide and critical application in many fields, such as photocatalysis, lithium-ion batteries, dye sensitized solar cells, sensing devices, functional ceramics, and light emitting devices [45,46,47,48,49]. With the development of SERS substrates from noble metals to semiconductor nanomaterials, ZnO nanostructures have tremendous potential to be exploited as active SERS substrates for the following reasons. Firstly, high-refractive-index ZnO has the ability to confine the light to enhance the SERS effect [50]. Secondly, abundant available morphologies of ZnO nanostructures are in favor of the combination with noble metals to improve the SERS activity. Additionally, the material’s many advantages, including bio-compatibility, tunable photoelectric properties, high chemical stability, superhydrophobicity, and photocatalytic self-cleaning effect [40,51,52] can be coordinated with the SERS effect on the ZnO nanostructure substrate to achieve versatility and multifunctionality.

However, an inferior EF (10–10^3^) is a fatal weakness of the pure ZnO nanomaterials, and has become a bottleneck in the development of semiconductor ZnO as an active SERS substrate. A top priority task is to find the root cause of the great disparity between the EF of noble metals and that of semiconductors. Two types of SERS enhancement mechanisms [53,54]—electromagnetic (EM) and chemical (CM)—have been studied, and although there are difficulties in clearly quantifying the specific contribution of EM and CM mechanisms to the EF [55], it has been revealed that EM can contribute 10–11 orders of magnitude to the EF of noble metals under special circumstances (i.e., “hot spots”), and another 10^3^ of EF comes from the CM [56,57]. With regard to semiconductors, the SERS enhancement is dominated by the CM, which usually has a value of 10–10^3^ [58,59]. After clarifying the primary cause of the weak enhancement of semiconductor substrates, two specific methods have been put forward to improve the SERS activity of semiconductor ZnO nanostructures, which are heavy elemental doping and combination of ZnO with noble metals.

## 2. Improved SERS Activity of ZnO Nanostructures: Theoretical Basis and Improved Methods

### 2.1. Theoretical Basis

EM and CM are two types of important enhancement mechanisms used to explain the SERS phenomenon on noble metals and semiconductors; thus, the SERS activity of semiconductor ZnO can be improved in two different ways: improving the electromagnetic enhancement, and improving the chemical enhancement. In order to make a great breakthrough in elevating the EF of semiconductor ZnO, efforts should be concentrated on improving the electromagnetic enhancement due to the enormous gap between the contributions of EM to noble metals (up to 10^11^) and semiconductors (little). Further, some progress should also be made in chemical enhancement due to its non-negligible contributions to the Raman enhancement.

#### 2.1.1. Theoretical Basis for Improving the Electromagnetic Enhancement of Semiconductor ZnO 

Here a pivotal issue should be raised: why is the electromagnetic enhancement of noble metals much larger than that of semiconductors? The local surface plasmon resonance (LSPR) [60] and “hot spots” effect in metallic nanoparticles may give the answers.

It is well known that the EM is the result of an enhanced local electric field generated by the collective resonance of surface plasmons in metallic nanoparticles under the irradiation of an incident laser. LSPR can occur in noble metal nanoparticles for the reason that the LSPR bands of noble metal nanoparticles are usually located in the visible (VIS) spectral region, which is the prerequisite for strongly absorbing the incident light by metallic nanoparticles. For semiconductors, the LSPR peak of the conduction band is normally centered in the near-infrared (NIR) spectral region due to the low electron density, while the LSPR peak of the valence band is approximately in the ultraviolet (UV) spectral region because of the high electron density (10^22^–10^24^ cm^−3^) [61,62], and consequently plasmons in semiconductors have scarcely any contribution to the EM effect, which is dominated by the LSPR under the visible light. Thus, the electromagnetic enhancement of semiconductors is far inferior to that of noble metals.

Additionally, when the gap between the metallic nanoparticles has a close spacing (less than 10 nm), the local electric field in these narrow junctions can get huge enhancement due to the coupling plasma resonance effect under the incident laser. These spots with an enhanced electric field are referred to as “hot spots” [63]. The comparative rarity of these “hot spots” notwithstanding, their remarkable contributions to the Raman enhancement can reach 10–11 orders of magnitude. The “hot spots” effect is usually achieved at the tips and corners on the rugged surface of noble metal nanostructures [64]. In conclusion, both the LSPR and the “hot spots” effect result in a remarkable Raman enhancement on noble metal substrates.

Through analysis of the aforementioned issue, an alternative solution is proposed to introduce electromagnetic enhancement into the semiconductor ZnO by means of tuning the LSPR peak location of semiconductor ZnO nanostructures near to the VIS spectral region and effectively creating “hot spots” in ZnO nanostructures.

**Figure 1:**
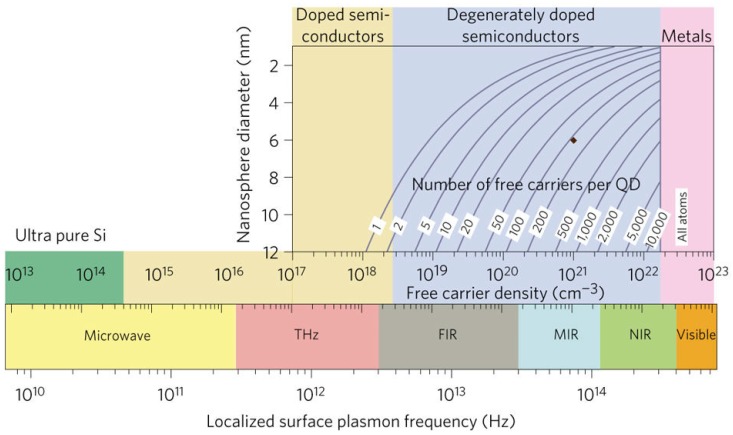
Local surface plasmon resonance (LSPR) frequency dependence on the free electron density for semiconductors, and number of free carriers per quantum dot (QD) required for nanosphere diameter ranging from 2 to 12 nm to achieve a free carrier density between 10^17^ and 10^23^ cm^−3^. Reproduced with permission from [65]. Copyright Nature Publishing Group, 2011.

Fortunately, the concrete influence of the free carrier density and nanosphere diameter, along with the number of free carriers per quantum dot (QD) on the LSPR frequency has been presented by Joseph M. Luther et al. in Figure 1 [65]. The LSPR frequency of semiconductors is primarily controlled by the free electron density. It can be conceived that the unified LSPR peak of ZnO nanostructures can be shifted to the NIR and even VIS spectral region when the free carrier concentration reaches up to 10^21^–10^23^ cm^−3^. It deserves the expectation that the EM can be introduced into semiconductors to improve the SERS activity by regulating the LSPR frequency with the doping level. The LSPR frequency can be calculated according to the equation:

(1) $$\omega_{p}={\left(\frac{N_{h}e^{2}}{\varepsilon_{0}m_{h}}\right)}^{1/2}$$

where $\omega_{p}$ is the LSPR frequency and $\varepsilon_{0}$ is the vacuum permittivity, $N_{h}$ is the density of free carriers (holes), $m_{h}$ is the hole effective mass, approximated as 0.8 $m_{0}$, where $m_{0}$ is the electron mass.

The Drude model [66] has been used by Xiangchao Ma et al. [67] to derive the critical carrier concentration required to make the real part of the permittivity of ZnO negative at 1000 nm according to the following equation:

(2) $$n>\frac{\varepsilon_{0}m^{*}}{e^{2}}\varepsilon_{b}\left(\omega_{c}^{2}+\gamma^{2}\right)$$

where n is the critical concentration, $\varepsilon_{0}$ is the permittivity of free space, e is the electron charge, $m^{*}$ is the carrier effective mass, $\varepsilon_{b}$ is the background permittivity which describes the polarization response of the core electrons, $\omega_{c}$ is chosen to be corresponding to the wavelength of 1000 nm which is relevant to photocatalysis and γ is approximated to be γ=eμm*; here, μ is the carrier mobility. For the semiconductor ZnO, the critical carrier concentration is calculated as being 1.62 × 10^21^ cm^−3^.

The effective creation of “hot spots” has as a means of enhancing Raman scattering, and then concerns should be paid to how to create “hot spots” on ZnO nanostructures. “Hot spots” on noble metals are usually located at the tips and corners, which can provide guidance for the morphology and structural optimization of ZnO nanomaterials. Randomly oriented rather than aligned nanowires (NWs), closely branched nanostructures instead of flat plate structure, and analogous ZnO nanostructures may generate more “hot spots” regions. Muhammad A. Khan et al. [50] have compared the SERS activity of randomly oriented ZnO NWs with partially aligned ZnO NWs to determine the contribution from the cross-junctions between nanowires. They found that the close coupling between these high-refractive-index ZnO NWs indeed improved the SERS activity.

At the same time, the relatively larger resolution limit of the conventional fabrication techniques compared to “hot spots” for metal nanomaterials, the easy aggregation among metallic nanoparticles, and the highly-uniform “hot spots” distribution are key challenges when creating “hot spots” between junctions of noble metals. Particular ZnO nanostructures can solve these problems to a certain extent thanks to their easily available steady nanostructures with crossings and junctions as well as their large plasmon-active surface to deposit and anchor noble metals. The branched ZnO nanostructures could be the backbone for the metallic nanoparticles and “hot spots”. For example, the close-coupling between the high-refractive-index ZnO nanowires can not only “trap” the incident light, but can also load metallic nanoparticles to create “hot spots” between these close metallic nanoparticles on the same or adjacent ZnO nanostructures. Muhammad A. Khan’s group [50] also deposited Au film on randomly oriented ZnO NWs, and the thickness of the Au film was confirmed to be vital to the Raman enhancement. They found that the moderate thickness—which was conducive to mutual surface plasmon interaction—can produce the strongest Raman enhancement due to the most appropriate gap to generate “hot spots” among the Au islands.

#### 2.1.2. Theoretical Basis for Improving Chemical Enhancement of Semiconductor ZnO

There is only a small difference between the contributions of the CM to noble metals and semiconductors, while its contributions to Raman enhancement cannot be neglected. It is considered that the CM is related to the interaction between molecules and substrates, and is dominated by the photo-induced charge transfer (PICT), which is thermodynamically permitted under the incident light excitation when the highest unoccupied molecular orbital (HOMO) and the lowest unoccupied molecular orbital (LUMO) of the molecules match with the conduction band and valence band of the semiconductors [68,69,70]. The CM cannot work if the incident laser does not have enough energy or if there is little interaction between the molecules and the substrate. Thus, the CM cannot take effect unless there is a laser with enough energy and a large active surface of ZnO nanostructures to adsorb the analyte.

When the size of the semiconductors becomes comparable to the size of the exciton Bohr radius, the exciton resonance cannot be ignored. The quantum confinement effect [71] can split the exciton levels and make the SERS spectra strongly depend on the size of semiconductor substrates. Compared with the noble metal, the quantum confinement effect of the semiconductor can elevate the EF by 10 [72]. Thus, a suitable size can improve the SERS activity of semiconductors to some extent via the quantum confinement effect. For ZnO nanostructures, the reported exciton Bohr radius are all less than 2.5 nm [73]. Nevertheless, the size effect does not work when the size of the ZnO nanostructures is much larger than the Bohr radius.

### 2.2. Improvement Methods 

Based on the theoretical analysis about the source of the huge gap between Raman enhancements of noble metals and semiconductors, two specific methods have been put forward to improve the SERS activity of semiconductor ZnO nanostructures, namely: heavy elemental doping and combination of ZnO nanomaterials with noble metals. Further, morphology, structure, and size optimizations are also important to improve the SERS activity of ZnO nanostructure substrates.

For heavy-doped ZnO nanostructures, the heavy doping degree is difficult to reach, and a giant free carrier density of 10^23^ is scarcely possible to obtain. Mg, Co, H, and some other elements have been doped into ZnO nanostructures, and the SERS activity can be improved to a limited degree. Certainly, it is also helpful to tune the LSPR peak location to the VIS region by decreasing QD sizes and increasing QD numbers according to the Figure 1.

With regard to another improvement method, there are three advantages to the use of noble metal/ZnO composite nanostructures as SERS substrates. Firstly, the excellent SERS activity of noble metal can help to improve the Raman enhancement of composite substrates by introducing the EM and “hot spots” effect into the ZnO nanostructures. Secondly, SERS activity can be combined with other properties of semiconductor ZnO to achieve the multifunctionality of composite substrates. Thirdly, noble metal/ZnO composite substrates can be more environmentally benign and chemically stable than noble metal substrates.

For noble metal/ZnO composite substrates, many detailed parameters can influence the SERS activity; for example, structural configurations of the composite substrate, shapes, sizes, relative location of noble metals and ZnO, etc. With regard to the structural configuration of the composite substrate, noble metal-deposited ZnO substrates and the reversed ZnO-coated noble metal substrates are different [74]. There are different interactions between the metal and ZnO, as well as different Raman enhancement mechanisms in these reversed substrates. For the reversed ZnO-coated noble metal substrate, the charge transfer CT between the semiconductor and molecules dominates the Raman enhancement. However, the noble metal plays a leading role in contributing to the SERS activity of the entire substrate when metallic nanoparticles are deposited on the ZnO nanostructures, and consequently the SERS activity is mainly influenced by shapes, sizes, and aggregation of the deposited metallic nanoparticles [75,76]. The LSPR peak location depends on the morphology, size, and composition of the metallic nanoparticles [76,77,78], and the number of “hot spots” depends on the aggregation of the metallic nanoparticles, which can be adjusted by distributing noble metals on ZnO substrates [79]. More specifically, for the noble metal Ag and Au, the characteristic LSPR peak of Ag nanoparticles (NPs) is usually centered on 390 nm, and that of Au NPs is usually located at a longer wavelength of 522 nm. Firstly, the LSPR peak position of the mixture of Ag and Au can be influenced by the molar ratios of Ag and Au, which has been well researched by Lakshminarayana Polavarapu et al. [77]. Secondly, the LSPR peak position of Au or Ag can be tuned by changing the morphology and size of the noble metal [78]. For instance, there are transversal and longitudinal LSPR for Au nanorods (NRs). When the Au NRs have a gradually decreased aspect ratio, the longitudinal LSPR will undergo a blue shift, while the transversal LSPR will undergo a red shift until they merge with each other into a single band. A controllable optical property and LSPR peak location can also be achieved by adjusting the size of metallic nanoparticles. “Hot spots” may generate when the metallic nanocrystals are aggregated, and it is critical to control the space between the nanoparticles to create “hot spots”. A novel and fantastic study concerning the temperature-controlled formation of “hot spots” has been done through depositing the metallic nanocrystals on a shape-thermoresponsive substrate [79], which can inspire us to creatively design a composite substrate with the ability to control the formation of “hot spots”, and consequently modulate the SERS activity.

## 3. A Unique Advantage of Semiconductor ZnO as SERS Substrate

However, a coin has two sides. It is not the absolute truth that the larger enhancement of the Raman signal means a better application of SERS technique, because the Raman scatterings of bands are usually enhanced selectively and non-uniformly in SERS. In some cases, extremely strong enhancement of a few bands may overshadow some weaker characteristic Raman peaks of “fingerprint regions”, which are useful to identify the desired analyte molecules. In spite of a comparatively weaker signal enhancement from the semiconductor ZnO than the noble metal substrates, a moderate enhancement of the Raman signal on the ZnO nanostructures could avoid the occurrence of signal masking and be in favor of the identification of all the spectral components. ZnO has been proven as a potential substrate to reveal some unnoticeable spectral components of the human whole blood with its moderate enhancement (20–30 fold) while ensuring that the original Raman spectrum information is not masked, and the SERS technique based on the ZnO substrate is promising to be a valuable tool to diagnose human diseases with human body fluids [80].

## 4. Necessity of Reporting a Meaningful Average EF 

The enhancement factor is one of the most important parameters used to characterize SERS performance. EF can be largely affected by many test conditions, such as the substrate types, target molecules, and excitation wavelength [24]. Two kinds of frequently used definitions and calculation methods are given below [81]. They respectively are the single molecule enhancement factor (SMEF) and a meaningful spatially average enhancement factor (average EF).

The SMEF is used to calculate the SERS enhancement of a given molecule in a specific position, so it is usually difficult to calculate the SMEF due to its precise definition. The SMEF is only suitable for theoretical estimation because of the difficulty in collecting the signal from just one molecule at a time. The single molecule enhancement factor is defined as the following:

(3) $$\mathrm{SMEF}=\frac{I_{\mathrm{SERS}}^{\mathrm{SM}}}{I_{\mathrm{RS}}^{\mathrm{SM}}}$$

where $I_{\mathrm{SERS}}^{\mathrm{SM}}$ is the SERS intensity of the single molecule and $I_{\mathrm{RS}}^{\mathrm{SM}}$ is the average Raman intensity per molecule under the same experimental condition. Only the single target molecule on the “hot spots” can be detected.

In many practical applications, the meaningful average EF is widely used to deal with the average SERS signal. It can be calculated by the following formula:

(4) $$\mathrm{EF}=\frac{I_{\mathrm{SERS}}/N_{\mathrm{SERS}}}{I_{\mathrm{bulk}}/N_{\mathrm{bulk}}}$$

where *I*_SERS_ and *I*_bulk_ are the integral intensity of Raman signals at the same peak position under SERS and target molecules conditions, respectively, *N*_SERS_ and *N*_bulk_ are respectively the average number of molecules adsorbed on the substrates in the scattering volume for the SERS and Raman (non-SERS) measurement.

At present, it is difficult and controversial to adopt a uniform EF to evaluate the performance of SERS substrates. Both of the above definitions can be used to calculate EF, and there is always a discrepancy between the single molecule EF and the average EF. To be objective, the SMEF is more precise and reasonable, while the averaged EF is more practical. Because the SMEF can greatly change from site to site due to the strong spatial locality of “hot spots”, the SMEF is usually used to accurately evaluate the contribution of one single molecule to the Raman enhancement. The meaningful spatially average EF is more practical for evaluating the performance of experimental substrates, while it is usually lower than the maximum SMEF and may underestimate the application of substrates with “hot spots”. Moreover, when the average EF is used for actual calculation, the accurate data is often required yet difficult to attain, and a reasonable hypothesis is always needed for the calculation. Thus, it should be realized that the averaged EFs can vary substantially between researchers due to diverse assumptions or inadequate description [82], and an appeal for a much more transparent determination of the performance of a given substrate is needed [63].

## 5. Synthesis of ZnO Nanostructures as SERS Substrates

### 5.1. Synthesis of 0-D ZnO Nanostructures

Zero-dimension (0-D) ZnO nanostructures usually include nanocrystals, nanospheres, and nanocages. Template, self-assembly, and thermal decomposition are three primary methods to prepare 0-D ZnO nanostructures.

#### 5.1.1. Synthesis of ZnO Nanospheres

The two-stage thermal decomposition method of the precursor [83,84,85,86,87] and the novel two-stage self-assembly method [88,89,90] are the most common fabrication methods for 0-D ZnO nanospheres. As one of the liquid phase methods, the thermal decomposition method has the advantage of easy acquisition of highly purified nanoparticles with uniform particle size. The self-assembly method has the superiority of forming a thermodynamically stable and structure-oriented nanostructure easily. In the two-stage thermal decomposition method of the precursor, the first stage is mixing and vigorously stirring the NaOH and zinc acetate (Zn(Ac)_2_) solution to produce the Zn(OH)_2_ precipitates, and then the NH_4_HCO_3_ powder is added to form the precursor of a small crystallite of Zn_5_(CO_3_)_2_(OH)_6_. In the second stage, the precursor is calcined at a high temperature to obtain the nanospheres. In the novel two-stage self-assembly method, monodisperse ZnO nanospheres can be derived by two-time precipitation at different temperatures. After the first ZnO precipitation is formed through the hydrolysis of zinc acetate dihydrate (Zn(Ac)_2_·2H_2_O) with the participation of diethylene glycol (DEG) under a higher temperature (typically 160 °C), the supernatant needs to be preserved by removing the first precipitation. The secondary reaction is performed in a similar way; a certain amount of the preserved supernatant is added to the solution before approaching a relatively low temperature (usually 150 °C), and then the ZnO colloidal spheres can be obtained.

#### 5.1.2. Synthesis of ZnO Nanocages

**Figure 2:**
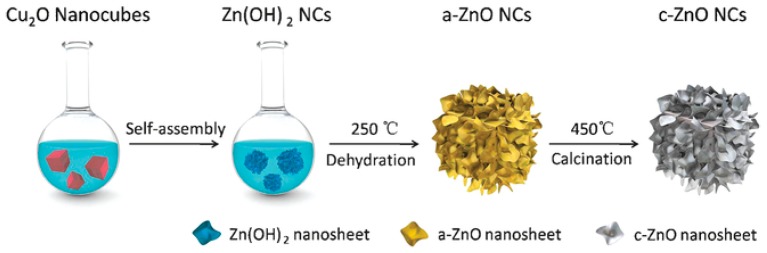
Schematic diagram of the synthesis of a- and c-ZnO NCs (amorphous and crystalline ZnO nanocages). Reproduced with permission from [91]. Copyright Wiley-VCH Verlag GmbH & Co. KGaA, Weinheim, 2017.

The ZnO nanocage can be prepared by a template method with a dehydration reaction. In order to synthesize the hollow amorphous ZnO nanocages, Xiaotian Wang [91] used the template method to synthesize the hollow Zn(OH)_2_ nanocrystals by employing Cu_2_O nanocubes as the structure-directing template and adding the stabilizing agent polyvinylpyrrolidone (PVP). Then, the Zn(OH)_2_ nanocrystals further underwent a dehydration reaction at 250 °C to form hollow amorphous ZnO nanocages (a-ZnO NCs). Crystalline ZnO nanocages (c-ZnO NCs) can be obtained by calcining the a-ZnO NCs. The specific synthetic schematic diagram is shown in Figure 2.

### 5.2. Synthesis of 1-D ZnO Nanostructures

**Figure 3:**
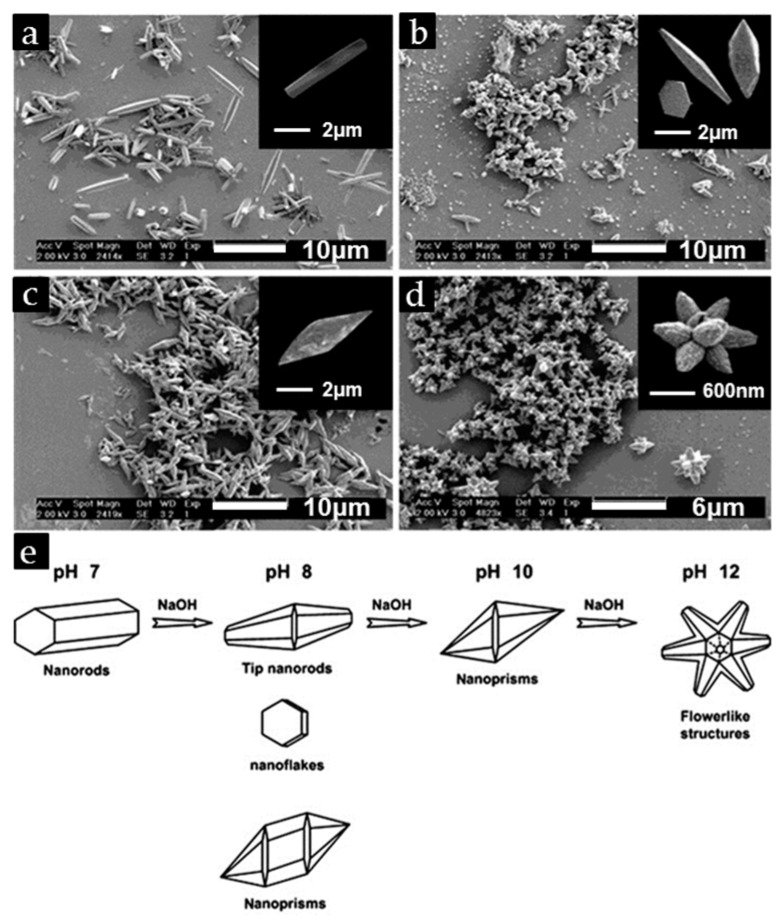
SEM images of ZnO deposited on Corning 7059 for a deposition time of 2 h and (**a**) pH 7, (**b**) pH 8, (**c**) pH 10, (**d**) pH 12, and the individual structures are exhibited in the insets of (**a**–**d**); (**e**) shows a schematic map of the morphology of the nanostructures. Reproduced with permission from [43]. Copyright Elsevier B.V., 2007.

Aqueous chemical growth (ACG) [92] is a simple method to deposit a 1-D ZnO nanostructure such as ZnO nanorods on the substrates. It is conventionally performed at a low temperature with the available inexpensive equipment. Many materials containing Corning 7059, indium tin oxide (ITO)-coated glass and silicon wafer can be used as the substrates for the deposition of ZnO NRs. In general, the Zn(NO_3_)^2+^ precursors, hexamethylenetetramine (HMTA) solution, and the substrate are heated at a constant temperature (typically 95 °C) in Pyrex glass bottles for a period of time to obtain ZnO NRs. pH has been adjusted by D. Vernardou [43] to get various ZnO morphologies ranging from the nanorods, tip nanorods, nanoprisms, to flower-like structures under pH of 7, 8, 10, and 12, respectively (Figure 3). Overall, ACG is a simple and practical method to deposit the 1-D ZnO nanostructures on substrates.

### 5.3. Synthesis of 2-D ZnO Nanostructures

#### 5.3.1. Synthesis of ZnO Nanosheets

**Figure 4:**
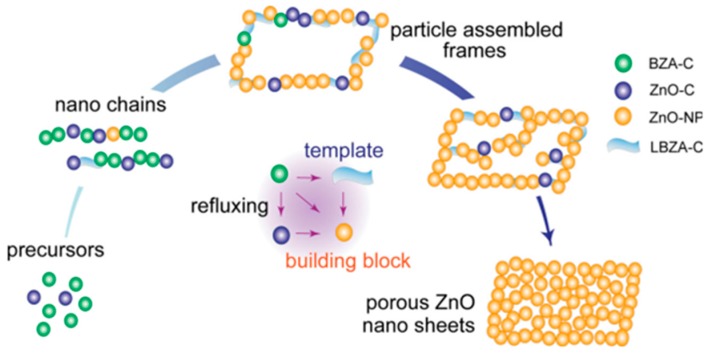
Schematic diagram of the composition and morphology evolution of the porous ZnO nanosheets. Reproduced with permission from [94]. Copyright WILEY-VCH Verlag GmbH & Co. KGaA, Weinheim, 2013. BZA-C: basic zinc acetate clusters; LBZA-C: layered basic zinc acetate clusters; ZnO-C: ZnO clusters; ZnO-NP: ZnO nanoparticles.

ZnO nanosheet structures are usually prepared by the template method. In a template method, the main body of the template is generally used as a configuration to control the shape and size of synthesized nanostructures. The most common template for porous ZnO nanostructure is the organic additives [93], which are likely to remain when finishing the oriented structure. Layered basic zinc acetate clusters (LBZA-C) as precursor—which can finally transform into the building blocks through refluxing—were creatively used as the template by Qian Liu [94] to assemble porous ZnO nanosheets in one pot (Figure 4). As the refluxing time went on, various evolving morphologies (nanochains, parallelogram frames, semi-filled nanosheets, and porous nanosheets) could be derived.

#### 5.3.2. Synthesis of ZnO Film

**Figure 5:**
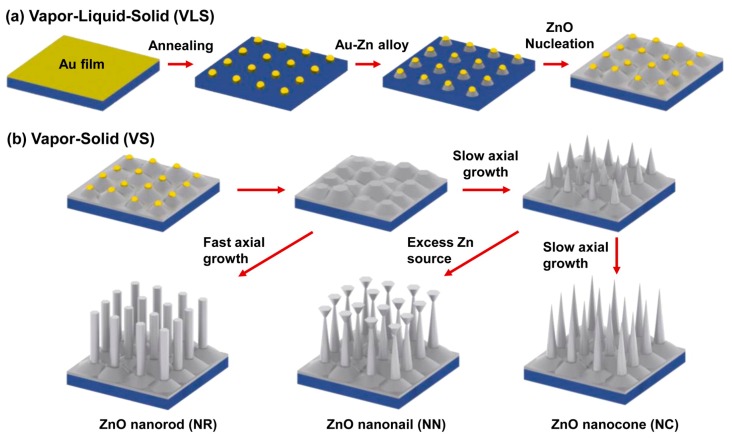
Schematic diagram of the growth mechanism of ZnO nanocones, nanorods, and nanonails. (**a**) Textured ZnO film formed with a vapor–liquid–solid (VLS) mechanism; (**b**) ZnO nanocones, nanorods, and nanonails grown with the vapor–solid (VS) mechanism. Reproduced with permission from [99]. Copyright American Chemical Society, 2015.

ZnO film is often synthesized on a substrate by the thermal evaporation process [95,96] in a horizontal quartz tube furnace. Evaporation source powder is usually placed in a tungsten, alumina, or quartz boat in the upstream of a flowing inert carrier gas such as argon gas, and the substrate is placed downstream near the evaporation source. After the oxygen in the furnace is thoroughly exhausted, the evaporation source will be heated at a temperature higher than the vaporization temperature, and the substrate is always maintained at a lower temperature for the growth of the film. ZnO film can be grown by this method using ZnO powder as the evaporation source. Naidu Dhanpal Jayram et al. [97] designed a vertical furnace to synthesize a worm-like Ag@ZnO thin film on a glass substrate through a similar thermal evaporation process. The substrate was at the top of the furnace, the ZnO powder and Ag wires in tungsten coil were at the bottom of the furnace as the evaporation source, and the evaporation was performed by setting up a current. A similar atomic layer deposition (ALD) method was used by Yufeng Shan et al. [98] to prepare wheatear-like ZnO nanostructures on Si substrate. Diethyl zinc and deionized (DI) water were respectively employed as the Zn precursor and the oxygen source. This thermal evaporation process was also used in the synthesis of ultra-sharp ZnO nanocone arrays deposited with the Au particle-on-Ag film systems by Youngoh Lee et al. [99]. Varied morphologies (ZnO nanorods, nanonails, and nanocones) (Figure 5) were derived by controlling the axial growth rate and the amount of the ZnO powder. Excessive ZnO powder was used for the growth of ZnO nanocones, and a fast and slow axial growth rate were respectively applied for synthesizing ZnO nanorods and ZnO nanocones.

### 5.4. Synthesis of 3-D ZnO Nanostructures 

#### 5.4.1. Synthesis of ZnO Nanorod Arrays

The microarray is one of the most representative ZnO nanostructures for SERS application because its distinguished structure can repeatedly scatter and further trap the light. Substrate materials are essential to the deposition of the ZnO microarrays. Electrodeposition [100] can be used to deposit the ZnO microarrays on the surface of the working electrode ITO glass by continuously bubbling oxygen with the electrodeposition solution of KCl and Zn(Ac)_2_.

**Figure 6:**
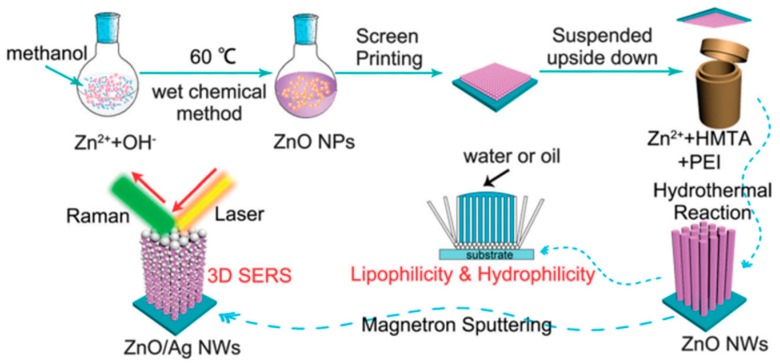
Schematic diagram of the preparation process of patterned ZnO nanowire arrays and the three-dimensional ZnO/Ag nanowire surface-enhanced Raman scattering (SERS) substrate. Reproduced with permission from [18]. Copyright The Royal Society of Chemistry, 2016. HMTA: hexamethylenetetramine; NW: nanowire; PEI: ethylene imine polymer.

**Figure 7:**
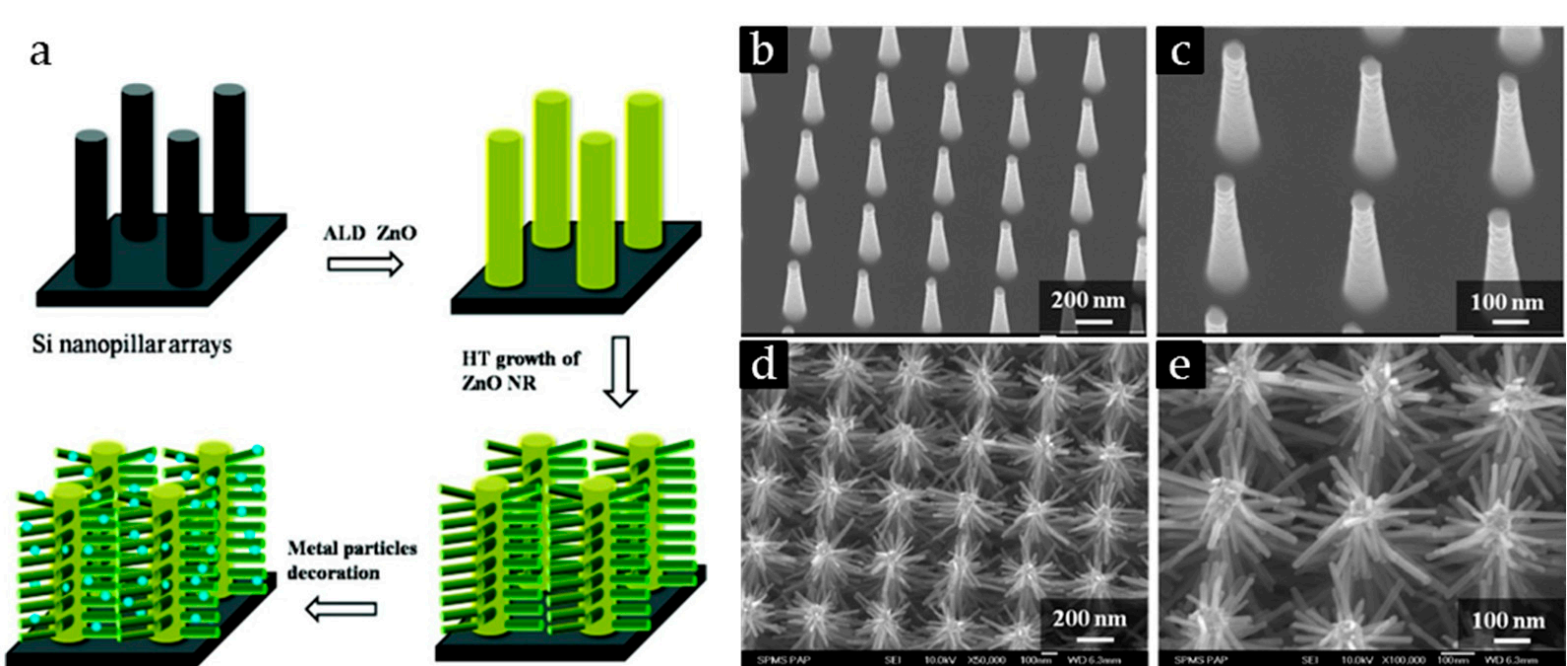
(**a**) Schematic diagram of the fabrication procedures for the three-dimensional ordered Si/ZnO nanotrees decorated by silver nanoparticles; SEM images of the (**b**) Si nanopillar arrays and the (**d**) ordered Si/ZnO nanotrees; as well as (**c**,**e**) the corresponding magnified SEM images. Reproduced with permission from [105]. Copyright American Chemical Society, 2010. ALD: atomic layer; NR: nanorod; HT: hydrothermal.

The three-step seeded growth process is another frequently used method to grow ZnO nanoarrays [101,102]. Firstly, a well-mixed steady ZnO nanocrystals sol is prepared [103] with KOH and Zn(Ac)_2_ in methanol or aqueous solution by stirring at 60 °C for 2 h according to a wet chemical method. Secondly, the sol is dropped or spin-cast many times on the surface of the substrate to form a layer of ZnO seed film, and the substrate materials can be ITO, silicon wafers, or plastic substrates. Then, the anneal operation is needed to ensure the adhesion of the ZnO nanocrystals on the substrate. Thirdly, a hydrothermal method is used to grow ZnO NR arrays on the surface of substrates with the addition of zinc nitrate hydrate and diethylenetriamine at a routine temperature of 90 °C or 95 °C. The previous two steps can also be replaced by a magnetron sputtering or atomic layer deposition method to coat a layer of ZnO seeds on the substrates. The zinc nitrate hydrate can also be substituted by zine acetate hexahydrate (Zn(Ac)_2_·6H_2_O). HMTA and methenamine can play the same role as the diethylenetriamine. The concrete preparation process of the patterned ZnO nanowire arrays is presented vividly in Figure 6 [18]. In addition, the ZnO nanorod and nanowire arrays can grow not only on plain substrate materials, but also on substrates with various kinds of backbones such as Si nanopillar and carbon-nanotube arrays [104]. Chuawei Cheng et al. [105] have successfully fabricated Si/ZnO nanotrees by growing the ZnO nanorod arrays on the Si nanopillar arrays by using the above method (Figure 7).

**Figure 8:**
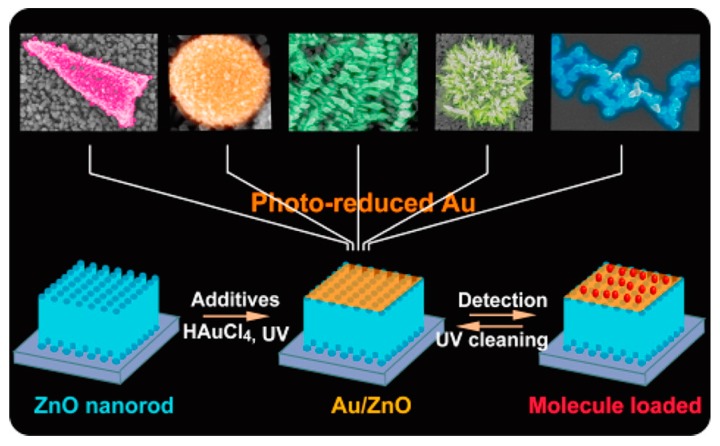
The photoinduced Au nanostructures in the form of conical Au, sphere Au, dendritic Au, sea-urchin-like Au, and Au chain from left to right, respectively. Reproduced with permission from [106]. Copyright American Chemical Society, 2016.

The synthesis of noble metal/ ZnO NRs composite nanostructures is primarily based on the above method for growing the ZnO NRs. Firstly, the ZnO NR arrays need to be grown on the substrates by the three-step seeded growth process. Then, the noble metal/ZnO composite will be completed with another two steps. The first step is to prepare the precursor solution for the growth of the noble metal. For various required morphologies of the noble metal, different additives are added to this solution. The next step is immersing the ZnO NR substrates into the precursor solution and then directly irradiating with UV light. Experimental parameters of the morphology-controlled Au/ZnO NRs composite have been studied by Jia-Quan Xu et al. [106]. The precursor solution is HAuCl_4_ aqueous solution, and the additives for the dendritic, sea-urchin-like, conical, chain-like, sphere-like Au (Figure 8) and Au NPs are, respectively, ammonium hydroxide (28%), phosphate-buffered saline (PBS), 0.15 M ammonium carbonate, 0.15 M *p*-phenylenediamine, 0.1 M HMTA, and saturated melamine (25 °C). Similarly, when decorating the Ag NPs on the ZnO NR arrays, AgNO_3_ solution is employed and NaBH_4_ solution usually needs to be added to reduce the adnexed Ag^+^ into Ag NPs on the surface of ZnO NRs with the wet chemistry method [107]. In addition, a series of methods including magnetron sputtering, thermal evaporation, or electron beam evaporation and photochemical deposition methods can also be used to deposit the noble metal on the ZnO NRs [18,105].

In recent years, a novel microfluidic technology has been on the rise and is rapidly becoming a new platform for sample preparation. It can manipulate the analyte flexibly and integrate with SERS to allow the instantaneous in-situ detection and investigation of analyte, even a single cell in the future. Yuliang Xie et al. [108] has grown 3-D Ag@ZnO nanostructure clusters by two sequential reactions catalyzed via an optothermal effect within the microfluidic devices. Firstly, ZnO NRs were grown on a gold-coated glass slide when focused by a continuous laser beam in the microfluidic channel containing Zn(NO_3_)_2_ and HMTA solution as precursors. Secondly, Ag NPs were grown on the ZnO NRs by focusing the laser beam onto the preformed ZnO NRs in the AgNO_3_ solution. It was very important to control the parameters of the laser (e.g., the heating power and the position of the focused laser spot) to determine the formation of ZnO NRs and Ag NPs.

#### 5.4.2. Synthesis of 3-D Sandwich Structure Assembly 

**Figure 9:**
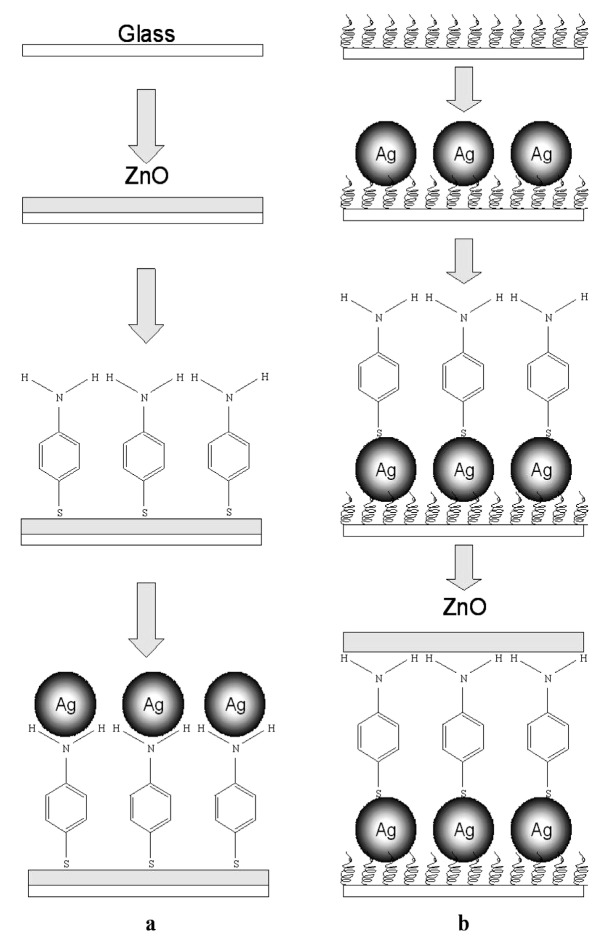
Schematic diagram of (**a**) ZnO/PATP (4-aminothiophenol)/Ag sandwich structure; (**b**) Ag/PATP/ZnO sandwich structure on glass substrates. Reproduced with permission from [110]. Copyright American Chemical Society, 2008.

The charge transfer in noble metal/molecule/semiconductor assemblies [109] is critical to the exploration of the chemical enhancement mechanism, thus it is necessary to summarize the assembly methods of the 3-D sandwich nanostructures. Here we take the assembly of the representative ZnO/PATP (4-aminothiophenol)/Ag and the reverse Ag/PATP/ZnO (Figure 9) as the example to introduce the fabrication method [110]. For the ZnO/PATP/Ag assembly, ZnO NR film is synthesized on the glass substrate with the above three-step seeded growth process, then the ZnO film is immersed into the PATP ethanol solution at room temperature for some time, and finally the obtained ZnO/PATP substrate is immersed into a silver colloid (which was prepared according to the literature [58]), to derive the ZnO/PATP/Ag assembly. The preparation process of the inverse Ag/PATP/ZnO assembly is similar to the former process. Note, however, that the glass cleaned by a mixed solution of H_2_O_2_ and H_2_SO_4_ should be immersed into a poly(diallydimethylammonium chloride) (PDDA) solution for the next self-assembly of Ag.

## 6. ZnO Nanostructures for Versatile SERS Application

In order to improve the SERS activity of ZnO nanostructures, the pure ZnO nanostructures are usually designed in varied shapes and sizes, doped with different elements and combined with noble metals. In this chapter, in addition to the three ZnO substrates above, three-dimensional sandwich (3-D-sandwich) structure nanomaterials as a distinctive ZnO composite materials are also introduced due to their unique 2-D stacked structures and the addition of typical 2-D materials such as graphene. Therefore, four types of ZnO nanostructures are used as the versatile SERS substrates: pure ZnO nanostructure materials, elemental doped ZnO nanomaterials, noble metal/ZnO composite nanomaterials, and 3-D-sandwich structure nanomaterials. Reported ensemble averaged enhancement factors on different ZnO nanostructure substrates are listed in Table 1, and they serve only as an indicator of SERS substrate performance.

### 6.1. Pure ZnO Nanostructure Materials

In 1996, Hao Wen et al. [30] successfully observed the surface-enhanced Raman signal of cyanine dye 1-methyl-1′-propylsulpho-2,2′-cyanine sulphonate (D266) molecules on pure ZnO colloids with an enhancement greater than 50. Semiconductor ZnO can exhibit SERS activity itself, but the enhancement is generally very weak. Many researchers have made a contribution to the design and fabrication of ZnO nanostructures with high SERS activities. Numerous microstructures have been devised and synthesized to realize the improvement of SERS activity of pure ZnO nanostructure materials, such as nanocrystals, nanospheres, nanowires, nanorods, nanoneedles, nanocones, nanosheets, nanocages, etc. SERS activities of these ZnO substrates are shape- or size-dependent due to different numbers of absorption sites for probed molecules, quantum confinement effect, multiple matter–light interactions in photonic microarrays, and optical cavity resonance by architectural configuration. An improved SERS performance has been achieved on a variety of pure ZnO nanostructures by many researchers.

#### 6.1.1. Morphology Optimization Design of Pure ZnO Substrates

Qian Liu et al. [94] synthesized 2-D parallelogram-shaped porous ZnO nanosheets with an enhancement of 10^3^ for 4-mercaptobenzoic acid (4-MBA) molecules. The porous morphology was beneficial to improving the SERS performance because it can provide large surface areas, abundant defects, and plentiful surface states, which can promote greater adsorption of probed molecules and favor efficient charge transfer, thus enhancing the SERS activity.

#### 6.1.2. Structure Optimization Design of Pure ZnO Substrates

In addition to the morphology optimization, special nanostructures with the ability to trap the light can also promote the Raman enhancement of pure semiconductors. A giant enhancement of the Raman signal from 4-mercaptopyridine (4-Mpy) adsorbed on 3-D ZnO nanoarray structures (nanowires and nanocones) was observed by Hae-Young Shin et al. [111]. They held the view that the CM dominated by the PICT between the substrates and the adsorbed molecules can only partly explain the enhancement, and that the great enhancement of SERS with EF of 10^3^ should be mainly attributed to the cavity-like resonance behavior in the well-constructed ZnO nanostructures, which has been confirmed by the finite-difference time-domain (FDTD) calculation. Their research provided a special way for us to design and employ structure-induced resonance to enhance the SERS activity.

#### 6.1.3. Size Optimization Design of Pure ZnO Substrates

Besides the nanostructures and morphologies of ZnO, particle sizes and measurement conditions also have an important impact on the SERS performance. The size-dependent SERS activity was explored by Zhihua Sun et al. [85], and they prepared ZnO nanocrystals with varied diameters in the range of 18–31 nm. The SERS effect was investigated by using the 4-Mpy and 4-MBA molecules as target molecules. They found that the size-dependence of ZnO nanostructure substrates was subsistent and the optimum particle size for the ZnO nanocrystals was nearly 28 nm. The CM dominated by the charge transfer between substrates and molecules was responsible for the size-dependent SERS activity, and they attributed the size-dependent charge-transfer resonance to the formation of a charge-transfer complex between a surface-bound exciton and the adsorbed molecules.

#### 6.1.4. Effects of Crystallinity on the SERS Activity of Pure ZnO Substrates

Recently, a novel study was carried out by Xiaotian Wang [91] with respect to the relationship between the SERS performance and the crystallinity of ZnO nanocages. It was worth noting that the amorphous ZnO nanocages (a-ZnO NCs) demonstrated a more excellent SERS activity than the crystalline ZnO nanocages (c-ZnO NCs). The difference of the lattice structure and crystallinity between a-ZnO NCs and c-ZnO NCs should account for this interesting finding. c-ZnO NCs had an ordered periodic lattice structure, which may strongly restrict the electrons and limit the escape and transfer of these electrons, while the long-range disordered amorphous lattice structure of a-ZnO NCs can lead the system to a metastable state, and make the charge transfer easier. Accordingly, the PICT process was facilitated and the polarization tensor was expanded, and the SERS activity was enhanced.

#### 6.1.5. Prerequisite for Realizing SERS on Pure ZnO Substrates: PICT

In order to achieve the prerequisite of realizing SERS in the 3-D ZnO NR arrays system, Xiaotian Wang et al. [100] investigated the SERS performance of 4-Mpy and 4-aminothiophenol (PATP) adsorbed on the ZnO NR arrays. They found that the efficient PICT between ZnO NRs and the probed molecules can amplify the probed molecules’ polarization tensor and the scattering cross-section, which was vital to the SERS enhancement, and therefore the effective PICT was the prerequisite for improving the SERS activity of semiconductor ZnO NR arrays. In addition, it was encouraging that the logarithmic concentration of pharmaceutical molecules ((Bu_4_N)_2_ [Ru(dcbpyH)_2_-(NCS)_2_] (N_719_) and acetaminophen, as well as the corresponding intensity of detected Raman peaks was linearly dependent. This finding provided feasibility evidence for tracing the photo-induced charges for the dye-sensitized solar cells (DSSCs), and there was promise for the exploitation of the semiconductor SERS substrates as chemosensors for pharmaceutical analysis.

Kwan Kim et al. [101] found a phenomenon that b_2_-type bands of 4-aminobenzenethiol (4-ABT) were absent in the 4-ABT Raman spectrum, whereas they were identified when adsorbed onto ZnO NR arrays, which was different from the ever-present a_1_-type bands. A similar phenomenon also emerged on 4-ABT derivatives including 4-(methylamino)benzenethiol (4-MABT), 4-(acetamido)benzenethiol (4-AABT), 4-(benzylideneamino)benzenethiol (4-BABT), 4,4’-dimercaptohydrazobenzene (4,4′-DMHAB) and 4,4’-dimercaptoazobenzene (4,4′-DMAB). The above evidence indicated that the b_2_-type band was related to the contact and interaction between the ZnO NRs and the probed molecules. To confirm this conjecture, they investigated the effect of the measurement conditions such as pH, excitation wavelength, and electric potential on the SERS signal. The results showed that the b_2_-type bands intensity would change with the pH because protonation of the amine group in the acidic solution made an increase of the LUMO level of 4-ABT, and thus the PICT process became harder and the signal became weak. The above discovery implied that b_2_-type bands were assigned to the PICT resonance. Similar evidence was also provided by the impact of excitation wavelength and electric potential on a_1_- and b_2_-type bands.

### 6.2. Elemental Doped ZnO Nanomaterials

Doping is a universal method used to introduce defects into semiconductors and change the lattice constant, the bond energy, and the energy gap of semiconductors. Appropriate doping element and concentration can promote the separation efficiency of the electron and hole, and improve the photocatalytic activity of semiconductors such as TiO_2_ and ZnO. Some studies have found that element doping can also be an effective means of enhancing the SERS activity, and the reason has been given as the following. For semiconductor substrates, the CM plays an important role in enhancing the Raman signal. When the probed molecules are chemisorbed on the ZnO nanostructures, surface defects which are introduced by the elemental doping will promote the formation of surface defects energy level, and make the CT process easier to process between the surface defects energy levels and the LUMO of the probed molecules with a relatively lower laser energy. It can be speculated that a higher concentration of dopant will result in a stronger SERS.

#### 6.2.1. Effects of Doping Concentration on SERS Activity of Elemental Doped ZnO Substrates

Xiangxin Xue et al. [83] studied the impact of Co-doping concentration on the SERS intensity with the system of 4-MBA molecules adsorbed on Co-doped ZnO NPs. Because the frequency of the LSPR of the semiconductors was far away from the laser wavelength, CT rather than LSPR was considered to be primarily responsible for the SERS effect. It was interesting that the optimum Co-doping concentration was 1% instead of a higher concentration, which may introduce more defects into the ZnO. It can be suspected that 1% Co-doping ZnO NPs had the largest possibility to generate the CT process. A reasonable explanation was provided that a higher defect concentration may cause the electron–hole recombination, which would compete with the CT from the Co-doping ZnO substrates to the molecules. A similar phenomenon was observed by Limin Chang et al. [84], who observed that there was an optimum Mg doping concentration of 3% when studying the SERS performance of the Mg-doping ZnO nanocrystals by using 4-MBA as probe molecules. Additionally, Szetsen Lee employed hydrogen and oxygen plasmas to introduce defects into hydrothermally synthesized ZnO NRs [92]. SERS activities can be promoted by controlling the concentration of defects with the help of H_2_ plasma to reduce the oxygen vacancy and the O_2_ plasma to increase the interstitial oxygen. In the meantime, the photoluminescence (PL) intensity of ZnO NRs can also be adjusted by the plasma treatment. A concordant combination of PL and SERS on the semiconductor ZnO nanostructures deserved further research.

### 6.3. Noble Metal/ZnO Composite Materials

Though ZnO nanostructures have many advantages and functionalities, their application as SERS substrates are always plagued by the inferior EF. It is understandable to combine noble metals with semiconductor ZnO nanostructures to achieve multifunctional and sensitive composite SERS substrates. There are two different structural configurations for noble metal/ZnO composite substrates, namely: metal-deposited ZnO substrate and ZnO-coated metal substrates. Ag is well-known for the skyscraping efficiency in enhancing the Raman signal, and is often used to combine with ZnO because the LSPR peak of Ag (390 nm) is adjacent to the UV absorption band (380 nm) of the nanoscale ZnO, which is favorable for a strong interfacial electronic coupling between the Ag and ZnO nanostructures. However, the life span of Ag is limited because it is easily oxidized. Compared with Ag, another common noble metal Au is more stable against oxidation, and the characteristic LSPR peak of Au is located at a longer wavelength of 522 nm [120]. For the biological Raman detection, a longer laser wavelength of 700–1100 nm with low scattering, absorption, and fluorescence is preferred [121,122], and thus the ability of better using the long wavelength laser by tuning LSPR of Au to the NIR region makes Au more biocompatible and extends the application of the SERS to biochemical, biomedical, and biological detection to revel the detailed information about DNA and proteins [108]. Nevertheless, Au NPs are always coated with a layer of chemical substance, which can help to avoid their aggregation and provide them with a functional surface chemistry. In fact, not only the aggregation of the metallic nanoparticles but also the adsorption of target molecules could be prevented by the protective layers. It is advisable to enhance their good points and avoid their shortcomings when using Au and Ag as the SERS substrates.

#### 6.3.1. Ag/ZnO Composite Materials 

**Figure 10:**
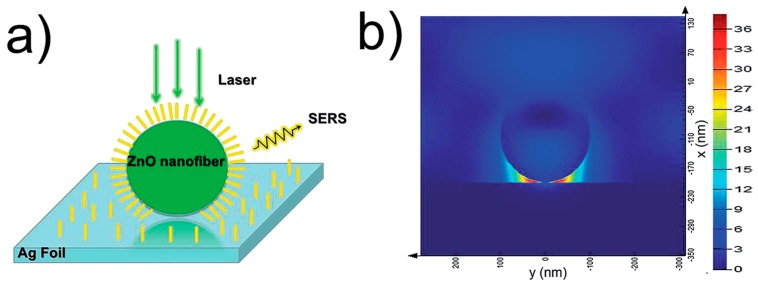
(**a**) Schematic diagram of ZnO nanofibers deposited on the surface of silver foil with probed molecules; (**b**) The distribution of the electric field for ZnO nanofibers deposited on the surface of silver foil calculated with finite-difference time-domain (FDTD) simulation. Reproduced with permission from [113]. Copyright The Royal Society of Chemistry, 2015.

ZnO nanostructures can not only be deposited on Ag foil, but can also be coated with Ag nanostructures. Wei Song et al. [113] attempted to deposit ZnO nanofibers on a silver foil surface to form a composite substrate. A high SERS intensity of the PATP (Figure 10a) was observed on this substrate, with an EF of 1.2 × 10^8^ and a detection limit down to 10^−12^ M. The enhanced scattering could be attributed to the EM arising from the exciton–plasmon interaction between ZnO nanofibers and the silver foil surface, which afforded the localization of the electric field at the gap between ZnO nanofibers and silver foil. It can be further demonstrated by simulating the distribution of the electric field for the system of the silver foil deposited by ZnO nanofibers with the FDTD (Figure 10b). Yufeng Shan et al. [98] fabricated wheatear-like ZnO nanoarrays decorated with Ag NPs, rhodamine 6G (R6G) was used as target molecules, and the EF reached up to 4.9 × 10^7^. Firstly, Ag NPs-deposited 3-D ZnO nanoarrays with a large surface area can generate a high-density of “hot spots”. Secondly, hydrogenation introduced many defects into ZnO and adjusted the surface energy band structure of the ZnO nanostructures, thus promoting charge transfer between the substrates and target molecules. Therefore, both the EM derived from the “hot spots” and the CM dominated by charge transfer, as well as the target molecules enrichment resulted from the large surface area of the ZnO nanoarrays, contributed to the SERS enhancement. Moreover, the appropriate hydrogenation degree increased the carrier concentration in ZnO nanostructures and evidently enhanced the photocatalytic activities in the meantime.

Apart from the photocatalytic performance, the super-hydrophobicity can also be integrated with SERS activity on the ZnO nanostructures. Naidu Dhanpal Jayram et al. [97] successfully prepared super-hydrophobic and high-sensitive worm-like silver-coated ZnO NWs with a contact angle of 163° and an enhancement of 3.082 × 10^7^ for detecting 10^−10^ M rhodamine 6G (R6G). The super-hydrophobicity was derived from the great increase of the air/water interface when the large fraction of air was entrapped in the interstices of the rough ZnO NWs. The sensitive SERS activity could be explained by the following two reasons. On the one hand, it was noteworthy that there was a strong interfacial electronic coupling between the neighboring ZnO NWs and Ag NPs. On the other hand, the higher contact angle indicated that the same amount of probed molecules in the droplet could be enriched in a smaller area, and consequently an increased surface coverage can be obtained, leading to an enhanced SERS signal on the substrate with high contact angle.

#### 6.3.2. Au/ZnO Composite Materials 

**Figure 11:**
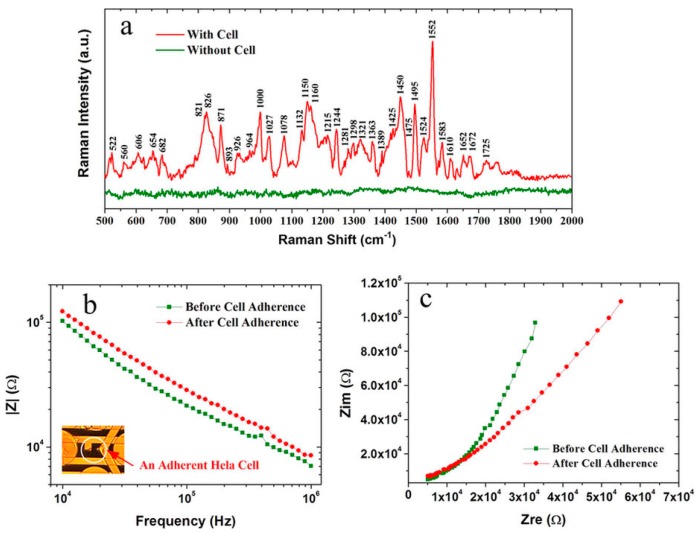
Dual-modal detection of a single Hela cell. (**a**) SERS spectrum of a single Hela cell attaching on the underneath electrode with Au nanostructure; (**b**) Comparison of cellular impedances before and after its attachment. Inset in (b) is the light microscope image of a single Hela cell adhering on the measuring electrodes; (**c**) Cole-Cole plots of the impedance measurements. Reproduced with permission from [116]. Copyright Nature Publishing Group, 2015.

In comparison to the Ag/ZnO composite nanostructures, Au/ZnO composite nanostructures are more biocompatible and can be applied in biological detection. Jiaquan Xu et al. [106] fabricated composite renewable biosensors with various Au nanostructures such as dendritic, spherical, sea-urchin-like, conical, and chain-like structures. Among these composite substrates, the dendritic Au NPs/ZnO composite exhibited the strongest Raman enhancement thanks to the hierarchical structure and the relative high coverage, which could provide abundant gaps for SERS enhancement, and thus even the 10^−9^ M R6G molecules can be detected by the dendritic Au/ZnO composite. The Au NPs/ZnO composite was not limited to the detection of NO released from the cells, it also had self-cleaning functionality, which can allow the substrates to be reused many times for the effective detection of target molecules as a renewable biosensor. Further, the in-situ SERS and electrochemical impedance spectroscopy (EIS) measurements were integrated in the Au nano-porous structure coated ZnO NRs to help us obtain the cellular information and monitor cell response to the environment (Figure 11) [116].

When combining a noble metal with ZnO nanostructures, the metallic nanoparticles are usually deposited on the surface of ZnO nanostructures while few studies pay attention to another reverse structural configuration, which is ZnO-coated metal nanostructures. For the metal-deposited ZnO nanostructures, the interaction between the metals and ZnO is often weak and the contribution of the substrate to SERS is mainly from the metal. Liping Liu et al. [74] have studied the flower-shaped Au-ZnO hybrid nanoparticles, which exhibited a stronger SERS signal of the b_2_ modes of PATP molecules because the CT from ZnO to molecules was enhanced by the additional electrons from Au NPs to the ZnO surface. In addition, the ZnO-coated Au substrates had a self-cleaning performance under visible light, and it can be developed into a promising SERS substrate simultaneously accompanied with the biocompatibility and visible-light-induced reproducibility.

#### 6.3.3. ZnO/Ag/Au Composite Materials 

**Figure 12:**
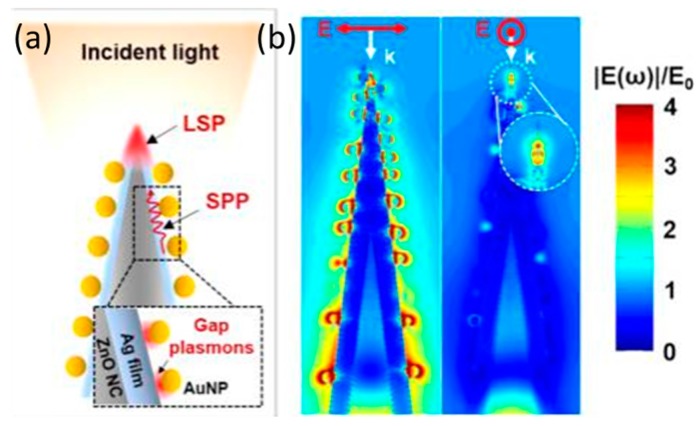
Ultrasharp ZnO nanocone arrays hybridized with the plasmonic systems of Au particle on Ag film (**a**) Schematic showing detection principle of fabricated SERS sensor; (**b**) Normalized electric field distribution for the ZnO/Ag/PDDA/AuNP NCs. Reproduced with permission from [99]. Copyright American Chemical Society, 2015. PDDA: poly(diallydimethylammonium chloride).

Structural configuration is also crucial for the SERS performance of the 3-D nanostructure. In order to achieve the ultra-high SERS activity with “hot spots” and the light trapping, a 3-D design of ultrasharp ZnO/Ag/Au NPs nanocone array was cleverly constructed by Youngoh Lee et al. [99]. The substrate even can detect target molecules at even zeptomole levels with the help of a large surface area provided by the 3-D ZnO nanostructures for the formation of SERS active sites, as well as the special metal nanostructures, which can act as efficient antennas and make the light absorbance increase. Further, the coupling of Ag film and Au NPs in this metal nanostructure can induce a large electric field at the particle–film gap, which has been theoretically evaluated and calculated by the discrete dipole approximation (DDA) method (Figure 12). The substrate was expected to be used for single-molecule detection at the zeptomole level.

### 6.4. 3-D-Sandwich Structure Nanomaterials

3-D-sandwich structure nanomaterials with a large contact area can usually exhibit an excellent SERS performance due to their particular structures. A majority of target molecules can be adsorbed by the large contact area of the 3-D-sandwich structure, and their particular structures are beneficial to the formation of “hot spots”. In addition, 3-D-sandwich structure can help the substrates to employ the distinctive properties of the constituent materials. The general constituent materials for 3-D-sandwich structures are noble metals, semiconductors, and typical 2-D layered materials such as graphene and MoS_2_. The relationship between the SERS performance and the structural configuration of the constituent materials is worthy of further research.

#### 6.4.1. Noble Metal/Molecules/ZnO 3-D-Sandwich Structural Composite Substrates 

The ZnO/PATP/Ag sandwich structure and its reverse Ag/PATP/ZnO sandwich structure were prepared by Zhihua Sun et al. [110]. It was discovered that these two substrates with the inverse structures showed the different SERS performance under a laser of 1064 nm due to the diverse connection of functional groups. It can be speculated that CM dominated by the charge transfer process primarily contributed to the SERS performance of these sandwich structures. The conjecture has been further confirmed by the evidence that the non-totally symmetric b_2_ modes of PATP were strongly enhanced in the ZnO/PATP/Ag sandwich structure, whereas in the reverse structure, they did not. Their discovery suggests that the charge transfer through the bridge-like interconnecting probed molecules between nanoscale metals and semiconductors may be detected by SERS. Based on the previous research, the relative intensity of non-totally symmetric modes can be considered as an indicator for the contributions of the charge transfer process to the SERS enhancement by Alexander P. Richter et al. [86]. After observing the size- and excitation wavelength-dependent charge transfer in the Ag/PATP/ZnO sandwich structure, they attempted to introduce a quantitative equation, as follows:

(5) $$P_{\mathrm{CT}}\left(k\right)=\frac{I^{k}\left(\mathrm{CT}\right){-I}^{k}(\mathrm{SPR})}{I^{k}\left(\mathrm{CT}\right){+I}^{0}(\mathrm{SPR})}$$

where $P_{\mathrm{CT}}\left(k\right)$ is the degree of charge transfer of a k-line and the k-line may be either a totally symmetric or nontotally symmetric line, *I*^k^(CT) is the intensity of a line where CT induced the increase of the SERS intensity caused by surface plasmon resonances (*I*^k^(SPR)), while *I*^0^(SPR) is the intensity of a line in the spectrum where only SPR contributed to the signal.

They also found that for a series of molecules with a low-lying unfilled π* orbital as an electron acceptor (PATP, 4-Mpy, and 4-MBA), the optimal particle diameter of ZnO was 27.7 nm for all wavelengths, which was consistent with the research results of Zhihua Sun et al. [110]. Through this study, they firmly ensured that the size-dependent SERS effect of the previous study was indeed the charge transfer in nature. Libin Yang et al. [117] considered that the Raman enhancement of PATP adsorbed on the Au/ZnO substrate was attributed to a CT contribution from the metal to molecules instead of the EM by examining the relative enhancement of the non-totally symmetric (b_2_) modes. According to the EM model proposed by Creighton [123] and Moskovits [124], the totally symmetric (a_1_) modes should have the strongest enhancement regardless of the orientation of the molecules. The selective enhancement of only the b_2_ mode among the a_1_, b_1_, and b_2_ modes cannot be explained by the EM mechanism. This series of studies about the relationship between the charge transfer and non-totally symmetric b_2_ modes provided us with an important understanding of the role of CM in SERS.

#### 6.4.2. Graphene/Noble Metal/ZnO 3-D-Sandwich Structural Composite Substrates 

Graphene is famous for a unique two-dimensional (2-D) layered structure with the exceptional electric, thermal, and optical properties [125,126], and has the potential to be exploited in the 3-D sandwich structure of composite substrates. In recent years, there have been studies about the possibility of using graphene as a SERS substrate [127], and graphene has been reported to have controllable SERS performance [128]. Many scientists have attempted to composite graphene with semiconductors and/or the noble metal nanostructures [129,130] in a layered sandwich form as the SERS substrates. These substrates can exhibit outstanding SERS activity, and the graphene has been confirmed to play an important role for the SERS enhancement. In the meantime, the participation of the graphene is also beneficial to improving the photocatalytic performance of the SERS substrates and making the SRES substrates more environmentally friendly.

**Figure 13:**
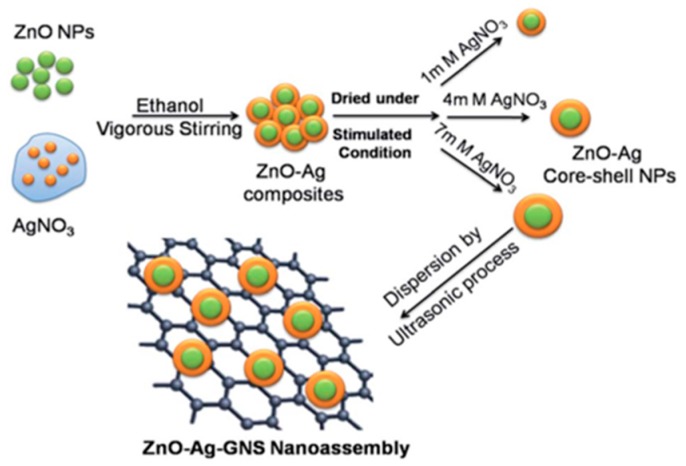
Schematic illustration of ZnO–Ag–graphene nanosheet (ZnO–Ag–GNS) nanoassembly synthesized by a wet chemical process. Reproduced with permission from [118]. Copyright The Royal Society of Chemistry, 2016.

Cheng-Chi Kuo et al. [131] have investigated the role that graphene played in the SERS and photocatalytic performance of the graphene–semiconductor (e.g., TiO_2_, ZnO) hybrid panel (GHP) substrates. They found that the precise number of graphene layers was critical to the performance of SERS and photocatalysis. Results showed that the hybrid with three layers of graphene (3L-GHP) possessed the maximum SERS performance, with an EF of 10^8^ when using R6G as the target molecule. Moreover, it also exhibited excellent photocatalytic activity when photodegrading the methylene blue (MB), due to the rapid electron and hole transfer through the graphene. R. Ajay Rakkesh et al. [118] constructed a ZnO–Ag–graphene nanosheet (ZnO–Ag–GNS) nanoassembly through integrating ZnO–Ag core–shell nanostructures on graphene nanosheets by a wet chemical process (Figure 13). The SERS activity of the ZnO–Ag–GNS nanostructure was enhanced by the LSPR of Ag NPs, as well as the easy interfacial charge transfer process due to the close contact of the graphene nanosheets with the metal Ag. This substrate can rapidly detect organic contaminants such as acridine orange dye (AO dye) and photodegrade the contamination simultaneously. This study gave us illumination that GNS was an excellent candidate for enhancing the SERS effect through accelerating the interfacial charge transfer process and enhancing the photocatalytic activity by means of reducing the recombination rate of the electron–hole pair. The graphene-based substrates have successfully exhibited SERS and photocatalytic activity simultaneously, and have expanded the application of SERS substrates.

Ya Chi Ko et al. [119] have fabricated Ag/ZnO/reduced graphene oxide (rGO) nanocomposite to detect bacteria by SERS and kill them in many ways. It can be realized by combining the photocatalytic property of ZnO NPs, the high specific surface area and NIR photothermal conversion property of rGO, as well as the bacteria-killing capability and SERS property of Ag NPs. *Escherichia coli* (*E. coli*) was successfully detected by this active Ag/ZnO/rGO substrate with a detection limit about 10^4^ cfu/mL, and was killed to different degrees by the substrates with or without the NIR or full Xe lamp irradiation.

## 7. Conclusions

In this review, we first discussed the development of SERS substrates from noble metals to semiconductors, and semiconductor ZnO was introduced as one of the potential SERS substrates. In order to improve the SERS activity of ZnO, the primary source of the great disparity between the EF of noble metals and that of ZnO nanostructures was analyzed and clarified, which can be attributed to the LSPR and “hot spots” of noble metals. Then, heavy elemental doping and the combination of noble metals with ZnO were put forward as the major improvement methods. Next, the preparation methods of varied ZnO nanostructures (0–3 dimensions) were summarized. Finally, we presented an overview of ZnO nanostructures for versatile SERS application. For pure ZnO nanostructures, the EF is usually 10^3^ due to the predominant chemical enhancement based on the PICT. With regards to the noble metals/ZnO composite substrates, the strongest EF can reach up to 10^10^–10^11^ with a detection limit at a zeptomole level of 10^−19^ M for the benzenethiol molecules by the ultrasharp ZnO/Ag/Au NPs nanocone substrate, which is promising for application in single molecule detection. In addition to the excellent SERS activity, many of these active ZnO nanostructure substrates are versatile; they not only can be used as chemosensors for detecting the NO released from the cells, but can also integrate EIS to obtain cellular information, identify “fingerprint regions” for disease by investigating human whole blood, and kill bacteria by photocatalysis.

However, there is little breakthrough in the improvement of SERS activity of semiconductors themselves. ZnO nanostructures are more likely to depend on the noble metals rather than themselves as the superior SERS substrates. Additionally, noble metal/ZnO substrates can still be plagued by the imperfection and instability problems during application. We look forward to elevating the SERS activity of ZnO nanostructures with the help of the optimization of shape, structure, and size, as well as sophisticated conformation of “hot spots”. A metalloid dielectric property of semiconductors can be expected to be realized in the future to greatly enhance the Raman scattering by tuning the LSPR peak near to the VIS spectra region. Further, the in-situ trace detection can be effectively and harmoniously integrated with other functionalities on the ZnO nanostructure substrates. In my view, there is an urgent need to discover and develop novel versatile semiconductors with ultra-high SERS activity, and this may be the most fundamental way to improve the SERS activity of semiconductors.

## Figures and Tables

**Table 1:** Reported ensemble averaged enhancement factors (EFs) on different ZnO nanostructure substrates. 4-MBA: 4-mercaptobenzoic acid; 4-Mpy: 4-mercaptopyridine; CM: chemical mechanism; D266: 1-methyl-1′-propylsulpho-2,2′-cyanine sulphonate; EM: electromagnetic mechanism; a-ZnO: amorphous ZnO; R6G: rhodamine 6G; rGO: reduced graphene oxide.

Substrates	Morphology	Probes	EF/Detection Limits	Mechanism	References
ZnO	Colloids	D266	More than 50	CM	[30]
ZnO	Nanocrystals	4-Mpy	10^3^	CM	[72]
ZnO	Nanoparticles	4-MBA & 4-Mpy	-	CM	[85]
ZnO	Nanowires, nanocones	4-Mpy	10^3^	“Hot spots” + cavity-like structural resonance	[111]
ZnO	Porous nanosheets	4-MBA	10^3^/10^−6^M	CM	[94]
ZnO	ZnO nanorod arrays sheathed with ZnO nanocrystals	4-Mpy	68	CM	[112]
a-ZnO	Nanocages	4-Mpy	6.62 × 10^5^	CM	[91]
Co-doping ZnO	Nanoparticles	4-MBA	-	CM	[83]
Mg-doping ZnO	Nanoparticles	4-MBA	-	CM	[106]
Ag/ZnO	Microspheres	4-Mpy	9 × 10^4^	EM + CM	[88]
Ag/ZnO	Wheatear-like ZnO nanoarrays decorated with Ag nanoparticles	R6G	4.9 × 10^7^	EM + CM	[98]
Ag/ZnO	Worm-like Ag-coated ZnO nanowires	R6G	3.082 × 10^7^ /10^−10^M	EM + CM	[97]
Ag/ZnO	ZnO nanowires deposited on an Ag foil surface	PATP	1.2 × 10^8^ /10^−12^M	EM + CM	[113]
Ag/ZnO	Urchin-like Ag NPs deposited on ZnO hollow nanosphere arrays	R6G	10^8^/10^−10^M	CM + “hot spots”	[114]
Ag/ZnO	Ag-nanoparticle-decorated Si/ZnO nanotrees	R6G	1 × 10^6^	EM + CM + structure- induced light trapping	[105]
Ag/ZnO	Ag nanoparticles deposited on ZnO nanowire arrays	Malachite green (MG)/amoxicillin	MG (2.5 × 10^10^ /10^−12^ M) Amoxicillin (10^−9^M)	“Hot spots”	[18]
Au/ZnO	Dendritic Au/ZnO composite	R6G	10^−9^M	EM + CM	[106]
Au/ZnO	Au-coated ZnO nanowires	4-methylbenzenethiol (4-MBT)/1,2-benzendithiol (1,2-BDT)	2.19 × 10^6^/ 4 × 10^5^M	EM + CM + “hot spots”	[50]
Au/ZnO	Flower-shaped ZnO-nanopyramids-coated Au core	PATP	-	CM of ZnO greatly excited by LSPR of Au core	[74]
Au/ZnO	Au-coated ZnO nanorods	MB	10^−12^M	“Hot spots”	[115]
Au/ZnO	Au nano-porous structure electroplated on ZnO nanorods	R6G	2.24 × 10^6^	“Hot spots”	[116]
ZnO/Ag/Au NPs	Ultrasharp nanocones	Benzenethiol (BT), R6G, adenine	10^10^–10^11^/BT (10^−19^ M), R6G (10^−17^ M), adenine (10^−17^ M)	EM + CM + “hot spots”	[99]
Au/ZnO/PATP	Layer-by-layer assembly	PATP	-	CM	[117]
ZnO-Ag- graphene nanosheets	Core–shell nanostructure integrated on nanosheets	Acridine orange (AO) dye	-	EM + CM	[118]
Ag/ZnO/rGO	Ag nanoparticles deposited on ZnO/rGO nanocomposite	*E.coli*	10^4^ cfu/mL	EM	[119]
